# Clustering of commercial fish sauce products based on an e-panel technique

**DOI:** 10.1016/j.dib.2017.11.083

**Published:** 2017-12-02

**Authors:** Mitsutoshi Nakano, Yoshimasa Sagane, Ryosuke Koizumi, Yozo Nakazawa, Masao Yamazaki, Kiyoharu Ikehama, Koichi Yoshida, Toshihiro Watanabe, Katsumi Takano, Hiroaki Sato

**Affiliations:** a Department of Food and Cosmetic Science, Faculty of Bioindustry, Tokyo University of Agriculture, 196 Yasaka, Abashiri, Hokkaido 099-2493, Japan; b The Organization for the Promotion of International Relationship, 709, 1-1-7 Motoakasaka Minato-ku, Tokyo 107-0051, Japan; c Alpha M.O.S. Japan K.K., 1-5-4 Takanawa, Minato-ku, Tokyo 108-0074, Japan; d Department of Applied Biology and Chemistry, Faculty of Applied Bioscience, Tokyo University of Agriculture, 1-1-1 Sakuragaoka, Setagaya-ku, Tokyo 156-8502, Japan

## Abstract

Fish sauce is a brownish liquid seasoning with a characteristic flavor that is produced in Asian countries and limited areas of Europe. The types of fish and shellfish and fermentation process used in its production depend on the region from which it derives. Variations in ingredients and fermentation procedures yield end products with different smells, tastes, and colors. For this data article, we employed an electronic panel (e-panel) technique including an electronic nose (e-nose), electronic tongue (e-tongue), and electronic eye (e-eye), in which smell, taste, and color are evaluated by sensors instead of the human nose, tongue, and eye to avoid subjective error. The presented data comprise clustering of 46 commercially available fish sauce products based separate e-nose, e-tongue, and e-eye test results. Sensory intensity data from the e-nose, e-tongue, and e-eye were separately classified by cluster analysis and are shown in dendrograms. The hierarchical cluster analysis indicates major three groups on e-nose and e-tongue data, and major four groups on e-eye data.

**Specifications Table**

**Table:** 

Subject area	Food science
More specific subject area	Food quality assessment using electronic sensor technology
Type of data	Table and dendrograms
How data was acquired	Each fish sauce product was tested with an e-nose (αFOX 4000, Alpha M.O.S., Toulouse, France), e-tongue (ASTREE, Alpha M.O.S.), and e-eye (IRIS VA300, Alpha M.O.S.), and the resulting data subjected to cluster analysis using R software.
Data format	Analyzed
Experimental factors	The fish sauce products used were made by various companies in Japan, Thailand, Vietnam, China, the Philippines, and Italy.
Experimental features	The smells, tastes, and colors of commercially available fish sauce products were assessed by e-nose, e-tongue, and e-eye analysis.
Data source location	Tokyo, Fukuoka, and Abashiri, Japan
Data accessibility	Data are presented in this article

**Value of the data**

• The availability of these data will enable a discussion of the influence of factors such as raw materials and the production process on smell, taste, and color development during fish sauce product manufacture.
• The data presented will allow estimation of fish sauce product consumer preferences in different countries.
• The smell, taste, and color data provided will be useful for culinary applications and the development of fish sauce products.

## 1 Data

**Fig. 1:**
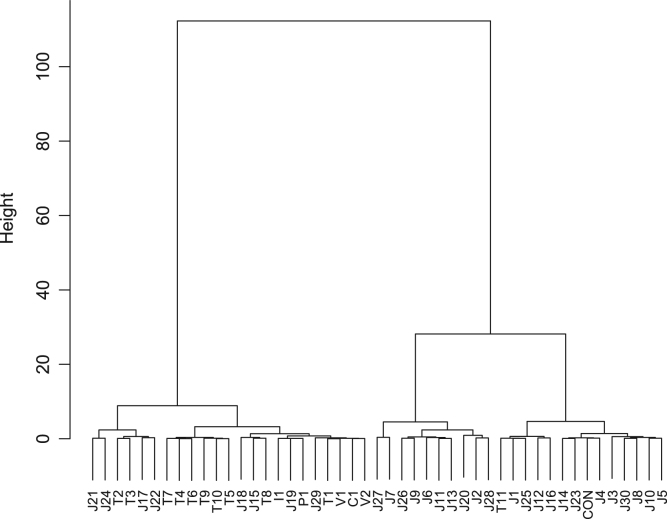
Dendrogram of fish sauce products based on the e-nose analysis.

**Fig. 2:**
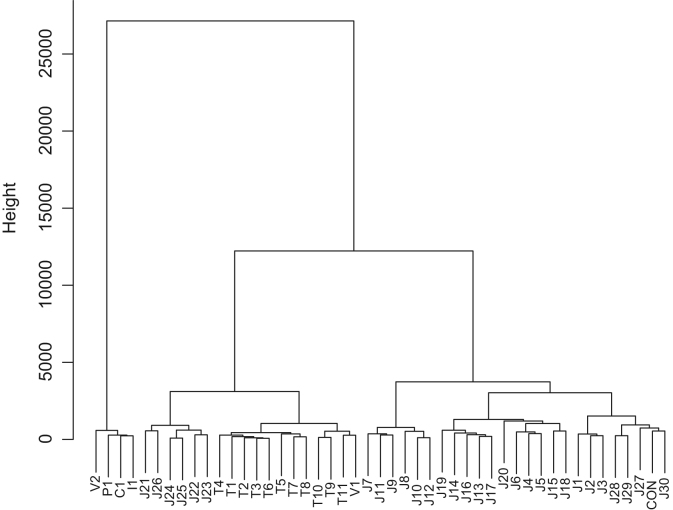
Dendrogram of fish sauce products based on the e-tongue analysis.

**Fig. 3:**
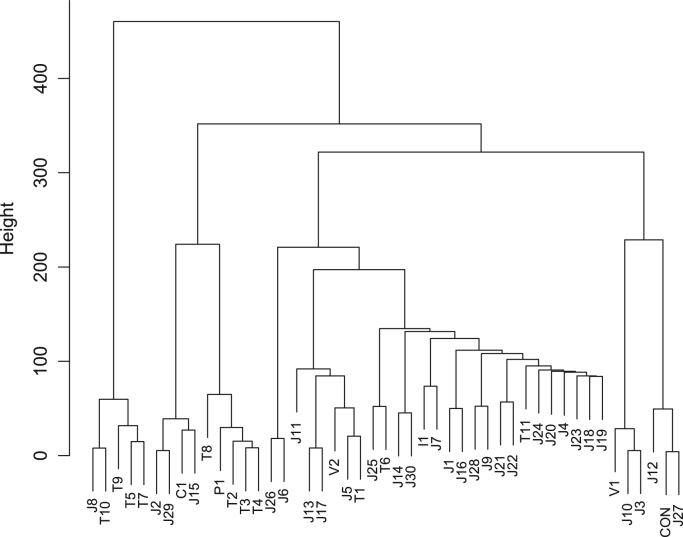
Dendrogram of fish sauce products based on the e-eye analysis.

Fish sauce is produced from seafood of various types. Table 1 displays the geographic origins and ingredients of the commercially available fish sauce products analyzed. Anchovy and sardine appear to be widely used in fish sauce production in Thailand and featured in fish sauce products from Vietnam, China, and Italy. A fish sauce product from the Philippines contained mackerel. Flying fish, which is often used as *dashi*, a broth employed in various dishes in Japan (especially in the *Kyushu* area), was found in several Japanese fish sauce products. Bonito is also employed for *dashi* preparation throughout Japan, and was also present in many of the fish sauce products tested from this country. In addition, Japanese sandfish, tuna, cod, sea bream, cutlassfish, deep-sea smelt, sea urchin, oyster, shrimp, and squid were found to be used in the manufacture of fish sauce products in Japan. Commercial fish sauce products from Japan, Thailand, Vietnam, China, the Philippines, and Italy clustered into three major groups when the e-nose data were analyzed (Fig. 1). Cluster analysis of the e-tongue data also identified three groups (Fig. 2), whereas that of the e-eye data yielded four major groups (Fig. 3).

**Table 1:** Fish sauce products used in this study.

Product ID	Origin	Ingredients
J1	Japan	Soy sauce, wheat, dried bonito shavings, kelp, and urchin
J2	Japan	Soy sauce, protein hydrolysate, saccharide, dried bonito extract, and fish sauce
J3	Japan	Soy sauce, sugar, mirin^a^, salt, dried bonito, and oyster extract
J4	Japan	Salt, mirin^a^, sugar, soy sauce, roasted flying fish, and alcohol
J5	Japan	Glucose-fructose, syrup, soy sauce, salt, dried bonito extract, mirin^a^, sugar, and roasted flying fish
J6	Japan	Soy sauce, fish, fermented seasoning, salt, glucose-fructose liquid sugar, and seaweed
J7	Japan	Soy sauce, sugar, glucose-fructose liquid sugar, vegetable protein hydrolysate, dried bonito extract, mirin^a^, bonito extract, salt, and yeast extract
J8	Japan	Salt, sugar, mirin^a^, soy sauce, roasted flying fish, fermented seasoning, roasted flying fish powder, and alcohol
J9	Japan	Soy sauce, saccharide, fructose liquid sugar, fermented seasoning, extract, honey, salt, roasted flying fish, and fish sauce
J10	Japan	Soy sauce, mirin^a^, sugar, salt, roasted flying fish, yeast extract, dried bonito flakes, kelp extract, and fish and shellfish extract
J11	Japan	Soy sauce, sugar, mirin^a^, salt, dried shrimp, brewed vinegar, yeast, extract, fish and shellfish extract, kelp extract, and dried shiitake mushroom
J12	Japan	Soy sauce, sugar, mirin^a^, dried bonito extract, salt, kelp extract, oyster extract, yeast extract, shiitake mushroom extract, and alcohol
J13	Japan	Soy sauce, sugar, roasted flying fish, salt, mirin^a^, yeast, extract, brewed vinegar, dried shiitake mushroom, kelp, and fish and shellfish extract
J14	Japan	Soy sauce, sugar, bonito extract, mirin^a^, salt, kelp extract, alcohol, and seasoning
J15	Japan	"Hata-Hata" Japanese sandfish and salt
J16	Japan	Soy sauce, sugar, fermented rice seasoning, bonito extract, dried anchovy extract, seasoning, and sweetener
J17	Japan	Soy sauce, saccharides, dried flying fish extract, salt, seasoning, sweetener, and alcohol
J18	Japan	"Hata-Hata" Japanese sandfish and salt
J19	Japan	*Acetes* shrimp and salt
J20	Japan	"Nigisu" deep-sea smelt, salt, soybeans, barley malt, and rice malt
J21	Japan	Squid, salt, rice malt, sake, and beer yeast extract
J22	Japan	Squid intestines and salt
J23	Japan	Squid intestines, salt, and shochu^b^
J24	Japan	Cod, barley malt, rice malt, salt, squid intestines, sugar, and fructose
J25	Japan	*Pagrus major*, salt, defatted soy bean meal, wheat, rice, and alcohol
J26	Japan	Flying fish, soybean, barley malt, rice malt, and salt
J27	Japan	Tuna, salt, soybean, barley malt, and rice malt
J28	Japan	Cutlassfish, salt, and rice malt
J29	Japan	Anchovy and salt
J30	Japan	Soy sauce, dried bonito extract, sugar, salt, yeast extract, amino acids, alcohol, caramel pigment, acidifier, acetic acid, and thiamine
T1	Thailand	Fish extract and salt
T2	Thailand	Anchovy, salt, and sugar
T3	Thailand	Anchovy extract, salt, sugar, and fructose
T4	Thailand	Sardine extract, salt, and sugar
T5	Thailand	Anchovy extract, salt, and sugar
T6	Thailand	Seafood, salt, and sugar
T7	Thailand	Sardine extract and salt
T8	Thailand	Seafood extract and salt
T9	Thailand	Sardine, salt, and sugar
T10	Thailand	Sardine extract, salt, and sugar
T11	Thailand	Fish sauce, soy sauce product, fructose, glucose-fructose liquid sugar, yeast extract, and amino acids
V1	Vietnam	Fish extract and salt
V2	Vietnam	Sardine and salt
C1	China	Anchovy, salt, and sugar
P1	Philippines	Mackerel
I1	Italy	Anchovy and salt
CON	Japan	Soy sauce as a control

a Rice wine predominantly used for cooking.

b Japanese spirit distilled from sweet potatoes, rice, etc.

## 2 Experimental design, materials and methods

### 2.1 Design

Fish sauce possesses distinctive smells, tastes and colors depending on the ingredient fish, fermentation process, geographic origins. Here we analyzed these features of fish sauces from Asian countries as well as Italian products. To avoid the effects of subjective assessment, we used an e-nose, e-tongue, and e-eye to precisely analyze the smell, taste, and color of fish sauce products from several countries. The e-nose and e-tongue consist of arrays of non-selective gas and liquid sensors with broad and partially overlapping selectivity towards the compounds present in a sample [1]. In addition, an e-eye was used to distinguish color components using camera-equipped apparatus and computer-assisted analysis. Of the commercial fish sauce products selected, 30 were produced in Japan, 11 were from Thailand, two derived from Vietnam, and one was from each of China, the Philippines, and Italy (Table 1). The list of numerical data indicating signal intensities on e-nose, e-tongue and e-eye sensors was brought to hierarchical cluster analysis to classify the fish sauces based on their smell, taste and color.

## 3 Materials and methods

### 3.1 Materials

All of the 46 fish sauce products were purchased at local markets in Tokyo, Fukuoka, and Abashiri (Japan). The ingredients of each product as listed on their labels are summarized in Table 1. For analysis, the products were assigned product IDs as follows: J1–J30 for the Japanese products; T1–T11 for the Thai products; V1 and V2 for the Vietnamese products; and P1, C1, and I1 for the Filipino, Chinese, and Italian products, respectively.

### 3.2 Evaluation of smell with e-nose analysis

E-nose analysis was performed with the αFOX 4000 smell analysis system (Alpha M.O.S., Toulouse, France), which has 18 metal oxide gas sensors for different selectivity patterns. Fish sauce product samples (0.5 g) were collected in 10-ml vials, placed in an autosampler, and analyzed under the following conditions: syringe temperature, 50 °C; oven temperature, 40 °C; injection speed, 2 ml/s. The analyzed data was obtained as numerical values of signal intensities.

### 3.3 Evaluation of taste with e-tongue analysis

E-tongue analysis was performed with the αASTREE taste analysis system (Alpha M.O.S.), which has seven liquid potentiometric sensors (SRS, GPS, STS, UMS, SPS, SWS, and BRS) and a reference electrode (Ag/AgCl). Fish sauce products diluted 21-fold were collected in a beaker and placed in an autosampler. Each sensor was immersed in the samples for 120 s at 20–25 °C with agitation to elicit a sensor response. A stable response value was then recorded at 120 s. The analyzed data was obtained as numerical values of signal intensities.

### 3.4 Evaluation of color with e-eye analysis

E-eye analysis of fish sauce products was carried out using an IRIS VA300 visual analyzer (Alpha M.O.S.) with a charge-coupled device camera. Five milliliters of fish sauce product was collected in a transparent plastic dish and placed in the measurement chamber. The color of the surface of each sample was measured three times. The collected color data were represented by IRIS color codes, which encompass 4096 colors. The analyzed data was obtained as numerical values of signal intensities.

### 3.5 Statistical analysis

Hierarchical cluster analysis was used to classify the fish sauces based on the smells, tastes and colors analyzed by e-nose, e-tongue and e-eye sensor sets. The datasets, comprising a series of sensor values from the e-nose, e-tongue, and e-eye tests, were subjected to cluster analysis by Ward's method using the program R-3.4.2 (http://www.R-project.org) [2].
